# A non-randomized trial of the effects of transcutaneous auricular vagus nerve stimulation on cognitive performance and resting state connectivity

**DOI:** 10.3389/fnhum.2026.1801066

**Published:** 2026-10-16

**Authors:** F. M. Filbey, C. Tyler Short, A. Leazure, N. E. Nekoui, T. Brown, S. Kroener, C. Liu

**Affiliations:** 1 The Department of Psychology, School of Behavioral Brain Sciences, University of Texas at Dallas, Richardson, TX, United States; 2 The Department of Neuroscience, College of Science and Mathematics, Tarleton State University, Stephenville, TX, United States; 3 The Department of Neuroscience, School of Behavioral Brain Sciences, University of Texas at Dallas, Richardson, TX, United States

**Keywords:** cognitive performance, cognitive training, EEG, neuromodulation, taVNS, Executive Function, BrainHQ

## Abstract

**Background:**

Transcutaneous auricular vagus nerve stimulation (taVNS) has shown promise for enhancing cognition and emotion regulation. To date, most human studies have focused on narrow learning paradigms that limit our understanding of how taVNS influences complex, integrative cognitive operations. Moreover, the underlying mechanisms of taVNS are still not well understood.

**Objective:**

Building on preclinical findings that vagus nerve stimulation enhances cognitive flexibility and memory in animal models, this study investigated whether acute taVNS can modulate executive function and neural connectivity in healthy adults.

**Methods:**

Using a quasi-experimental design, forty healthy participants received either active or sham taVNS while completing executive function tasks and adaptive cognitive training (BrainHQ). High-density electroencephalography (EEG) was recorded during stimulation to assess cortical activity and functional connectivity across frequency bands.

**Results:**

Multivariate analyses demonstrated significant effects of taVNS on cognitive function and EEG. *Post-hoc* group comparisons showed that compared to sham stimulation, active taVNS was associated with improved BrainHQ performance and decreased delta-band connectivity in left frontal, central, and parietal regions. Delta-band connectivity during active taVNS was positively associated with pre-to-post improvements in mood. No difference in executive function tasks was found between active and sham taVNS. Sensitivity analyses using adjusted models showed that these effects were sensitive to age, sex, and education.

**Conclusion:**

By evaluating multiple dimensions of executive function, these exploratory findings suggest that active taVNS may influence selected aspects of executive performance and frontal–parietal delta connectivity, potentially reflecting enhanced neural efficiency arising from the interaction between vagally mediated neuromodulation and continuously adaptive cognitive demands. However, these effects did not survive multiple-comparison correction and should therefore be interpreted cautiously and replicated in larger, prospectively powered studies.

## 1 Introduction

In recent years, non-invasive neuromodulation via transcutaneous auricular vagus nerve stimulation (taVNS) has emerged as a promising tool to modulate cognitive processes, particularly executive function (Ridgewell et al., 2021). For example, taVNS has recently been shown to improve inhibitory control by increasing intrinsic prefrontal couplings (Zhu et al., 2024). taVNS engages the afferent vagal pathway, projecting from the auricular branch of the vagus nerve to the nucleus tractus solitarius in the brainstem, which in turn activates the locus coeruleus (LC) and basal forebrain (Butt et al., 2020; Chen and Liu, 2025). Activation of these nuclei triggers the release of norepinephrine (NE) and acetylcholine, two neuromodulators critical for arousal regulation, synaptic plasticity, and cognitive control (Aston-Jones and Cohen, 2005). This pathway provides a mechanism to link peripheral stimulation with central neural changes observable in human electrophysiology. taVNS has been shown to modulate cortical oscillatory dynamics, particularly in low-frequency bands (delta and theta), and enhance functional connectivity within fronto-parietal and salience networks, which are essential for executive control (Camargo et al., 2025; Han et al., 2024; Monti et al., 2025). Reduced low-frequency band connectivity during active vagus nerve stimulation (VNS) has been interpreted as increased neural efficiency, that may reflect more focused, task-relevant processing consistent with LC-NE–mediated cortical desynchronization (Vespa et al., 2021; Sangare et al., 2020).

Executive functions (cognitive flexibility, inhibitory control, and working memory) are sensitive to vagal tone and network integrity (Thayer and Lane, 2000; Thayer et al., 2009). Because of this sensitivity, pairing taVNS with tasks targeting these functions may enhance cognitive control, and by extension, may facilitate clinical processes such as extinction learning and abstinence in substance use disorders. Preclinical research supports this translational link. Childs et al. (2019) showed that when pairing extinction training with VNS, the VNS-induced release of neuromodulators facilitates and consolidates the synaptic plasticity that results from training alone. Nazari et al. (2025) found that taVNS in rats ameliorated the impairment of working and recognition memory induced by morphine and provided anxiolytic effects. Human neuroimaging research similarly demonstrates that both invasive and transcutaneous VNS produces acute and sustained changes in fronto-parietal, default mode, and salience networks, regions implicated in attention, emotion regulation, and cognitive control (Keatch et al., 2023). Together, these findings suggest that taVNS can enhance executive function via modulation of brain connectivity and neuromodulator systems. Moreover, cognitive training research suggests that improving executive functions may in turn influence vagal control of the autonomic nervous system, indicating a bidirectional relationship between central cognitive networks and peripheral autonomic regulation (Thayer and Sternberg, 2009).

Despite promising findings, studies to date show only small to moderate effects of taVNS on overall cognitive performance in healthy adults, with inconsistent protocols complicating the interpretation. Moreover, existing taVNS studies have predominantly employed narrow learning paradigms that provide limited insight into how taVNS influences complex, integrative cognitive operations. These task-specific designs restrict generalizability, obscure underlying mechanisms, and fail to capture network-level modulation of executive control processes that are critical for adaptive, real-world behavior. In the present study, we translated preclinical findings of vagus-mediated cognitive enhancement into a controlled human model that assesses multiple domains of executive function. We conducted a single blind, sham controlled, nonrandomized design study in healthy adults to test whether acute taVNS influences executive function performance, mood states, and underlying neurophysiological markers (EEG connectivity, especially low-frequency bands). We hypothesized that taVNS would enhance performance on executive-control tasks and modulate neural oscillatory connectivity (particularly in delta band) in frontal–parietal networks.

## 2 Methods

This study was approved by the University of Texas at Dallas Institutional Review Board, and all participants provided informed consent.

### 2.1 Participants

Forty-two healthy volunteers (21 females, 21 males, mean age = 25 years, range 18–54) were recruited from the general community through social media posts, physical flyers, and undergraduate psychology courses between April 2022 – June 2023. For this study, participants were eligible if they were (1) aged 18–55, (2) proficient in English, (3) right-handed, and (4) able to give their informed consent. Exclusion criteria included: (1) history of brain injury or neurological diagnoses (including loss of consciousness for >6 min in past 6 months), (2) Axis I disorder including any substance use disorder, (3) currently taking psychotropic medications, and (4) EEG and taVNS contraindications – pregnancy, implants, cardiac disorders (autonomic nervous system disorders, e.g., orthostatic hypotension, bradycardia, problems with breathing and swallowing), ongoing pharmacological treatment.

The target sample of approximately 40 participants was informed by the scale of exploratory human taVNS studies available when the study was designed, which typically enrolled approximately 30–60 participants to examine acute cognitive, physiological, and neuroimaging outcomes (e.g., Borges et al., 2020; Sun et al., 2021), and by the practical feasibility and resources available for completing the combined taVNS, cognitive-training, EEG, and behavioral protocol.

### 2.2 Study procedure

A sham-controlled, nonrandomized design was used where participants were blinded to the taVNS condition (Figure 1). Participants were asked to complete a single study visit and were compensated for their time. Forty-two participants were assigned to either the active (*n* = 21) or sham (*n* = 21) condition using an alternating allocation procedure based on enrollment order, stratified by age and sex to promote balanced representation across groups (Table 1).

**Figure 1:**
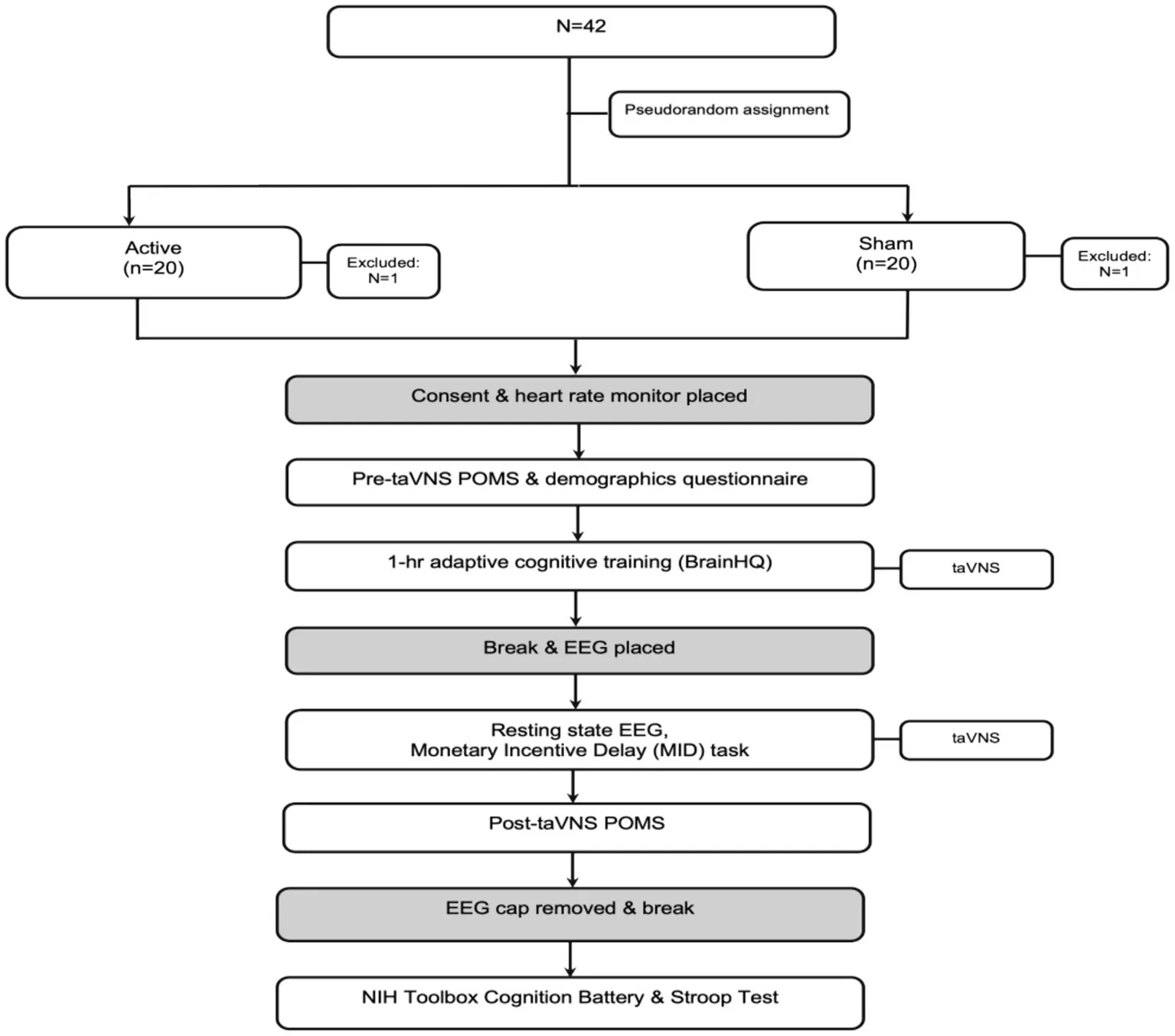
Study procedure.

**Table 1:** Sample characteristics.

	Active (*n* = 21)		Sham (*n* = 21)		Total (*n* = 42)	
	*n*	%	*n*	%	*n*	%
Sex						
Male	11	52.38	10	47.62	21	50
Female	10	47.62	11	52.38	21	50
Ethnicity						
Hispanic or Latino	2	9.52	3	14.29	5	11.9
Race						
Asian	13	61.9	10	47.62	23	54.76
Black	1	4.76	2	9.52	3	7.14
White	5	23.81	8	38.1	13	30.95
Other race	2	9.52	1	4.76	3	7.14
Employment						
Employed (full time)	3	14.29	3	14.29	6	14.29
Employed (part time)	2	9.52	9	42.86	11	26.19
Unemployed (voluntarily)	11	52.38	5	23.81	16	38.1
Unemployed (involuntarily)	5	23.81	4	19.05	9	21.43
Income						
<$20,000/yr.	4	19.05	4	19.05	8	19.05
$20,000–$39,999/yr.	3	14.29	4	19.05	7	16.67
$40,000–$59,999/yr.	2	9.52	1	4.76	3	7.14
>$60,000/yr.	12	57.14	12	57.14	24	57.14
Education						
H.S. Graduate/GED	10	47.62	5	23.81	15	35.71
Associate’s	1	4.76	2	9.52	3	7.14
Bachelor’s	9	42.86	13	61.9	22	52.38
Master’s	1	4.76	1	4.76	2	4.76
Age	25.24 (M)	9.5 (SD)	25.57 (M)	7.49 (SD)	25.4 (M)	8.56 (SD)

The experimental procedure is illustrated in Figure 1. Following informed consent, participants were fitted with a heart rate monitor and completed a demographics questionnaire and the Profile of Mood States (POMS) (McNair et al., 1971). Following the questionnaires, participants were fitted with the taVNS device according to manufacturer guidelines. Participants then completed 1 h of cognitive training with simultaneous taVNS (active or sham). Immediately after cognitive training, participants completed the taVNS Side Effects Questionnaire to assess stimulation-related sensations and tolerability. After the cognitive training session, participants were given a 15-min rest break to minimize fatigue and residual stimulation effects. The EEG cap was then applied, and the taVNS device was reattached. Participants then underwent simultaneous taVNS and EEG recording during rest with eyes closed (2 min), rest with eyes open (2 min), and while performing the Monetary Incentive Delay (MID) task. Immediately after completing the MID, the taVNS Side Effects Questionnaire was re-collected to assess any post-stimulation symptoms. A post-taVNS POMS was also collected to evaluate potential mood changes associated with stimulation. Lastly, following another fifteen-minute break, participants completed the NIH Toolbox Cognition Battery (executive function measures) and a Stroop Color Word task (without taVNS). All data were collected while the participants were seated.

### 2.3 HRV acquisition

Because heart rate variability (HRV) may be an indicator of acute vagal efferent activity of the cardiac branches (Thayer and Lane, 2000; Thayer et al., 2009), we recorded single-lead electrocardiogram data using the Polar H10 chest strap device (Polar Electro UK Ltd., Warwick, United Kingdom). Recordings were collected continuously during data collection with participants seated and breathing normally. We used the Polar H10 based on validation by Hinde et al. (2021), identifying it as the most accurate consumer device for HRV analysis, and additionally supported by Schaffarczyk et al. (2022). HRV was automatically processed using the Elite HRV application. The application’s proprietary processing algorithm identifies and corrects artifacts in the R–R interval series before calculating the root mean square of successive differences (RMSSD), a time-domain measure of beat-to-beat variability. RMSSD was then natural-log transformed (lnRMSSD) and converted to the application’s standardized 0–100 HRV score, with higher values indicating greater beat-to-beat variability.

### 2.4 Mood assessment

We used the Profile of Mood States (POMS) (McNair et al., 1971) to evaluate mood pre- and post-taVNS to determine the potential effects of taVNS on mood states. POMS is a 65-item 5-point Likert scale that provides a measure of six dimensions of mood: tension, depression, anger, vigor, fatigue, and confusion. Difference scores (post-session POMS score subtracted from pre-session POMS score) were generated for each dimension, and an overall index of negative mood, the Total Mood Disturbance (TMD) score, using the standard formula:

$$\mathrm{TMD}=(\begin{array}{l} \text{Tension}+\text{Depression}+\text{Anger}\\ +\text{Fatigue}+\text{Confusion} \end{array})-\text{Vigor}$$

### 2.5 taVNS procedure

We used the NEMOS^®^ taVNS neurostimulation device (tVNS Technologies, previously Cerbomed) that delivers stimuli (pulse width of 200–300 μs at 25 Hz; 30 s on and 30 s off) to the auricular branch of the vagus nerve located in the left cymba concha (Sönmez et al., 2025). Stimulation parameters were chosen in correspondence with recommended guidelines (Farmer et al., 2021) and are consistent with published experimental designs (Badran et al., 2018; Frangos et al., 2015; Yakunina et al., 2017). In the active condition, the stimulation electrodes were applied to the cymba concha in the left ear. Similar to previous studies, the stimulation electrodes were placed on the center of the left ear lobe in the sham condition (Kraus et al., 2007; Van Leusden et al., 2015). Stimulation intensity (mA) was set to just below the pain threshold for each participant (active mean = 1.3 mA; sham mean = 1.8 mA; combined mean = 1.6 mA). The pain threshold was determined using a gradual ascending-intensity procedure, in which stimulation intensity was incrementally increased from a low level until participants reported the sensation as painful. The taVNS Side Effects Questionnaire was collected to assess stimulation-related sensations and tolerability following stimulation. All items were assessed on a Likert scale from 1 (Strongly disagree) to 5 (Strongly agree).

### 2.6 Cognitive training

Cognitive training via Brain HQ through Posit Science^1^ was used during both active and sham taVNS conditions. BrainHQ is an adaptive, computer-based cognitive training program designed to assess and improve executive functions, including processing speed, attention, and cognitive flexibility. BrainHQ uses an algorithm that continuously adjusts task difficulty based on participant performance, whereby improved performance increases task difficulty and lower performance decreases task difficulty. Each task had a required level of performance to proceed to the next task. We used the following BrainHQ tasks for cognitive training: Mind Bender, Divided Attention, and Mixed Signals. Mind Bender is a set-shifting task that trains participants to discriminate between target images and distractor images. Divided Attention is an attention task that trains participants to discriminate specific criteria in two images while ignoring competing details. Mixed Signals is based on the Stroop task, which trains participants to match auditory and visual stimuli. The tasks were presented in a fixed sequence that lasted for 1 h (Figure 1).

### 2.7 Cognitive assessments

A modified monetary incentive delay (MID) task was collected to measure anticipation and response to positive and negative incentives (Knutson et al., 2000). The task consisted of 40 reward trials, 40 punishment trials, and 20 neutral trials. During the task, participants pressed a keyboard key within a sliding timescale in order to receive a reward or avoid a punishment across low ($0.20) and high ($5.00) magnitudes. Participants were incentivized to perform well on the task by being told they would keep the earnings they accumulated during the task. Using a dynamic algorithm, most participants received $20–35. The reaction time per trial was recorded. The total duration of this task was ~11 min.

The NIH Toolbox Cognition Battery (Version 2) was completed on an iPad with the instructions and practice trials prior to each task. Specific cognitive tasks were selected to capture the three core components of Miyake et al.’s (2000) tripartite model of executive function: cognitive flexibility (shifting), inhibitory control, and working memory (updating). While the NIH Toolbox Cognition Battery structurally separates Working Memory into a distinct subdomain for scoring purposes (Weintraub et al., 2013), the developers explicitly designed the battery to include measures of these three aspects identified in the tripartite model (Zelazo et al., 2014). Consequently, we assessed cognitive flexibility using the Dimensional Change Card Sort Test, inhibitory control using the Flanker Inhibitory Control and Attention Test, and working memory using the List Sorting Working Memory Test. All three measures have been validated for use in adult populations (Zelazo et al., 2014; Tulsky et al., 2014).

The Stroop Color Word task used in this study was modified from the Stroop task provided with PsychoPy version 2022 (Peirce et al., 2019) and consisted of 60 trials. Participants responded with their pointer finger on the “left”, “up”, and “right” keys replaced with the “R”, “G”, & “B” keys, using the down key as a home base between responses.

### 2.8 EEG acquisition and preprocessing

Resting state EEG was recorded using ANT Neuro Waveguard Original 64 channel cap, ANT Neuro eego™ mylab amplifier, & eego™ system recording software version 1.9.2. The electrodes were placed using the international 10–20 system and recorded with a sampling frequency of 1,000 Hz. The reference electrode was placed on the left mastoid and re-referenced offline to both left and right mastoids. The channel impedances were kept below 7kΩ. EEG was recorded in the sitting position during the eyes closed (EC) and eyes open (EO) resting state condition for 2 min each.

The EEG signals were band-pass filtered at 0.1–55 Hz to reduce low-frequency drifts and high-frequency muscle noise and eliminate line noise at 60 Hz, using a 4th-order infinite impulse response (IIR) Butterworth filter, after DC removal. Bad channels exhibiting flat or excessively noisy signals were visually identified and reconstructed using spherical interpolation. Independent component analysis (ICA) with 63 components and “ICLabel” functions were used to automatically classify independent components into brain, eye blinks and movements, cardiac and muscle activity, channel and line noise, or other with probabilities (Pion-Tonachini et al., 2019). Visual inspection based on scalp topography, time course and activity power spectrum, was performed to reject artifactual independent components. Epochs were two seconds long with 50% overlap. Pre-processing of the recorded EEG signals was performed in EEGLAB 2023.1 (Delorme and Makeig, 2004) and further analyses (spectral power and coherence) were conducted using custom scripts written in MATLAB R2023a (MathWorks, Natick, MA).

Spectral power was calculated for all EEG channels using fast Fourier transforms (FFT) using Welch’s power spectral density estimate and applied in the delta (1–≤4 Hz), theta (4–<8 Hz), alpha (8–12 Hz), beta (13–30 Hz), and gamma (31–50 Hz) bands. Relative power was calculated as a percentage of the total power (1–50 Hz) for each frequency band. The EEG channels were grouped into eight regions corresponding to the major cortical areas: frontal (F), central (C), parietal (P), and occipital (O) in the left (L) and right (R) hemispheres. The midline channels (i.e., Fp, Fz, FCz, Cz, Pz, Poz, and Oz) were excluded from the analyses.

Spectral coherence between pairs of electrodes in specific frequency bands was calculated to assess functional connectivity (i.e., statistical dependence of signals) among cortical regions (Rubinov and Sporns, 2010; Babiloni et al., 2016) as defined by each electrode. Coherence quantifies the amount of synchrony of electrophysiological signals with respect to their frequency and amplitude (Fries, 2005). To determine differences in cortical communication between cortical regions, intrahemispheric and interhemispheric coherence was calculated. The mean coherence values from all possible pairs of electrodes between two corresponding regions represented the interhemispheric coherence. The mean coherence values from all possible pairs of electrodes between P, C, O, and F regions were used to calculate intrahemispheric coherence within the left and right hemispheres. A similar approach to measure coherence has been reported previously (Prashad et al., 2018). The clustering coefficient quantifies what fraction of a region’s connections are also connected and quantifies connectivity around specific regions (Farahani et al., 2019).

### 2.9 Statistical analyses

Analyses were performed using Python (SciPy Stats).

Demographic characteristics were compared descriptively to characterize the study groups. An independent-samples *t*-test was conducted to test age differences between groups. Mann–Whitney U tests were conducted to test between-group differences in education and income. Differences in nominal categorical variables (sex, country of birth, marital status) were analyzed using Pearson’s Chi-square tests (chi^2^). For categorical variables with sparse data or expected cell counts <5 (employment, ethnicity, race), categories were collapsed into binary outcomes (e.g., employed vs. unemployed; white vs. non-white) and compared between groups using Fisher’s Exact Tests. All statistical tests were two-tailed with significance defined as alpha <0.05.

For our primary aim of testing the effects of taVNS on executive function, we conducted a multivariate analysis of variance (MANOVA) using statsmodels Python library on the executive function outcome measures: MID reward reaction time, MID punishment reaction time, age-corrected scores for NIH Dimensional Change Card Sort score, age-corrected NIH Flanker Inhibitory Control and Attention score, age-corrected NIH List Sorting Working Memory score, Stroop interference reaction time (congruent minus incongruent), and BrainHQ performance. To determine whether there was a difference between active and sham groups in spectral power, interhemispheric coherence, intrahemispheric coherence, and clustering coefficient in all bands during the EC and EO resting state conditions, we conducted a one-way MANOVA. Covariates were specified *a priori* based on theoretical relevance and prior evidence identifying them as predictors of executive-function and EEG outcomes. Accordingly, age, sex, and education were included in the adjusted models regardless of whether the groups differed significantly on these variables.

Secondary analyses were performed to determine if taVNS altered mood. We analyzed the change in POMS sub-domain scores (post-stimulation minus pre-stimulation) using a one-way ANOVA with group (active vs. sham) as the between-subjects factor. Additionally, we examined taVNS effects on cardiac vagal activity. We analyzed InRMSSD-derived HRV scores using a linear mixed-effects model specifying fixed effects for group, timepoint, and their interaction, with a random intercept for subject.

Significance for *post-hoc* univariate analyses was determined using Bonferroni correction.

## 3 Results

### 3.1 Study sample

No significant differences were observed between the active and sham groups across any demographic variables (all *p* > 0.05). The groups were matched on age (active M years = 25.2, sham M years = 25.6; *p* = 0.903), as well as education (*p* = 0.189) and income (*p* = 0.856). Categorical comparisons revealed no significant differences in sex (*p* > 0.99), country of birth (*p* > 0.99), marital status (*p* = 0.328), ethnicity (*p* > 0.99), race (*p* = 0.332), or employment (*p* = 0.058). Because the groups did not differ significantly on these variables and their causal role was uncertain, the adjusted model is presented as a sensitivity analysis rather than as the sole basis for inference.

One taVNS active participant (male) was excluded from analyses due to technical issues during the BrainHQ training. An additional participant (female in sham condition) was excluded because of poor EEG recording. Hence, analyses included *N* = 40 (20 active/20 sham).

There was a significant difference in self-reported neck pain from the Adverse Side Effects Scale (*p* < 0.05) where the sham group (1.38 ± 0.74) reported more neck pain than the active group (1.00 ± 0.00).

### 3.2 Effects of taVNS on cognitive performance

The unadjusted analysis suggested a multivariate effect of taVNS on executive function measures, Wilks’ Λ = 0.66, *F*(7, 33) = 2.39, *p* = 0.043, partial η^2^ = 0.34, 95% CI [0.00, 0.49] (Figure 2). However, this effect was attenuated in the covariate-adjusted primary model, which did not provide clear evidence of an independent group effect.

**Figure 2:**
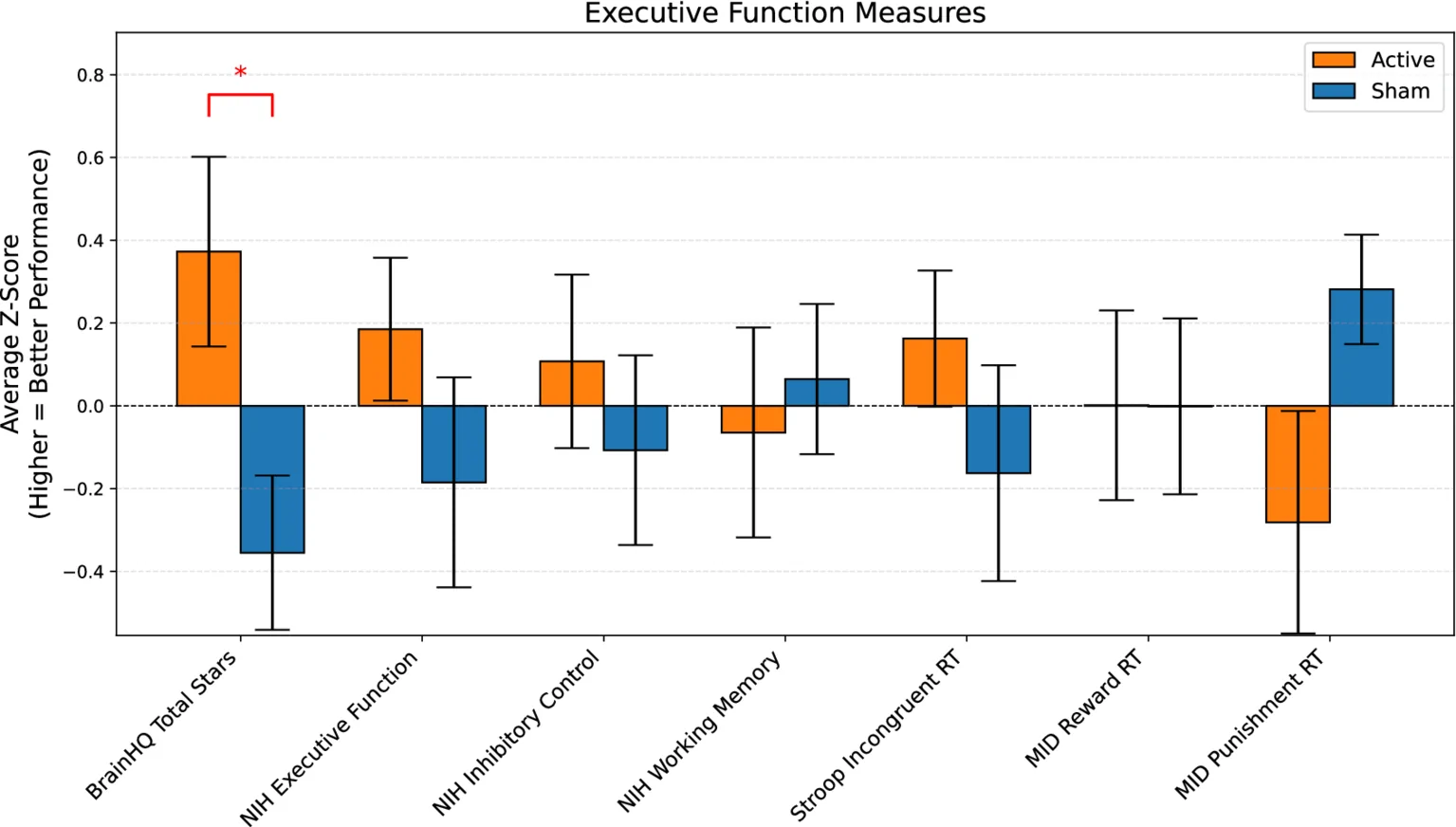
Effect of taVNS on executive function measures. Difference between taVNS active and sham conditions on executive function measures. MANOVA results showed a significant effect of taVNS condition on executive function. *Post hoc t*-test showed significantly greater BrainHQ performance in the active vs. sham condition. Scores are presented as mean standardized Z-scores ± SEM. Higher Z-scores indicate better executive control across all domains: better performance on brain training or executive function tests, reduced interference (Stroop), and greater mobilization of cognitive resources in response to incentives (MID). A one-way MANOVA indicated a significant multivariate effect of stimulation condition (Active vs. Sham) on executive function performance (Wilks’ Λ = 0.66, *F*(7, 33) = 2.39, *p* = 0.043, η_p_^2^ = 0.34). Follow-up independent samples *t*-tests revealed that the active group demonstrated significantly higher performance on the BrainHQ task compared to sham (*t* = 2.47, *p* = 0.018). No significant differences were observed for the NIH Toolbox scores, Stroop interference, or MID reaction time measures. * *p* < 0.05.

Follow-up univariate analyses revealed a nominal effect of group on BrainHQ total stars (*F*(1, 39) = 6.11, uncorrected *p* = 0.018, Cohen’s d = 0.77, 95% CI [0.13, 1.32]), though this did not survive the Bonferroni correction. No other dependent variables showed significant group differences (reward-neutral RT, punishment-neutral RT, inhibitory control, working memory, executive function, and Stroop interference).

### 3.3 Effects of taVNS on resting state EEG

The unadjusted analyses showed that taVNS had an effect on spectral coherence. This association was attenuated after adjustment for age, sex, and education, indicating that the finding was sensitive to covariate specification.

*Post hoc* univariate tests indicated that, during the eyes-closed (EC) condition, the active group had lower delta-band clustering coefficients than the sham group in the left frontal, *F*(1, 38) = 4.80, *p* = 0.035, partial η^2^ = 0.11, 95% CI [0.00, 0.32]; left central, *F*(1, 38) = 4.30, *p* = 0.046, partial η^2^ = 0.10, 95% CI [0.00, 0.30]; and left parietal regions, *F*(1, 38) = 4.20, *p* = 0.047, partial η^2^ = 0.10, 95% CI [0.00, 0.30]. Directionally similar, but more uncertain, effects were observed in the left occipital, *F*(1, 38) = 3.80, *p* = 0.059, partial η^2^ = 0.09, 95% CI [0.00, 0.29]; right central, *F*(1, 38) = 3.20, *p* = 0.082, partial η^2^ = 0.08, 95% CI [0.00, 0.27]; right parietal, *F*(1, 38) = 3.10, *p* = 0.087, partial η^2^ = 0.08, 95% CI [0.00, 0.27]; and right occipital regions, *F*(1, 38) = 3.70, *p* = 0.063, partial η^2^ = 0.09, 95% CI [0.00, 0.29]. None of these effects met the Bonferroni-adjusted significance threshold (α = 0.00125). The estimated effect in the right frontal region was smaller and uncertain, *F*(1, 38) = 2.40, *p* = 0.131, partial η^2^ = 0.059, 95% CI [0.00, 0.25]. No clear group differences were observed in the theta, alpha, beta, or gamma bands during the EC condition, or in any frequency band during the eyes-open condition (Figure 3).

**Figure 3:**
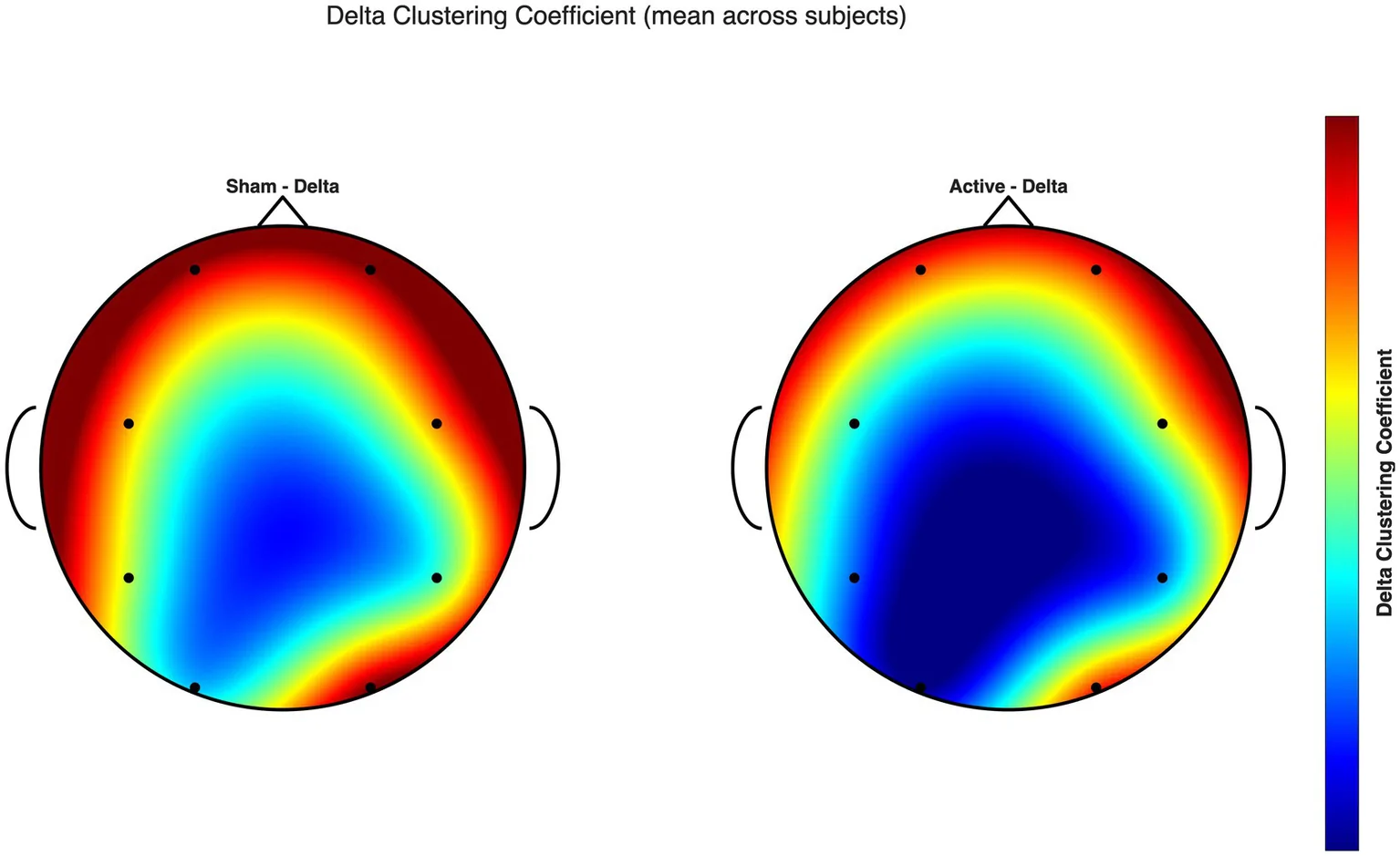
Mean scalp distributions of the delta-band clustering coefficient in the Sham and Active groups. Interpolated topographic maps show lower clustering coefficients in the Active group than in the Sham group, with the largest reductions over the left frontal, central, and parietal regions. These regional differences were statistically significant (left frontal: F(1,38) = 4.80, *p* = 0.035; left central: F(1,38) = 4.30, *p* = 0.046; left parietal: F(1,38) = 4.20, *p* = 0.047). Directionally similar but nonsignificant reductions were observed in the left occipital, right central, right parietal, and right occipital regions. Colors represent relative clustering coefficient magnitude, with warmer colors indicating higher values and cooler colors indicating lower values. Black dots denote electrode locations.

Analyses showed no significant effect of taVNS on EEG relative power or spectral coherence.

### 3.4 *Post-hoc* tests: correlations between BrainHQ, POMS domains and clustering coefficient

Given the group difference in cognitive performance and EEG clustering coefficient, we tested the relationship between BrainHQ performance and delta band clustering coefficient. No significant correlations emerged.

Given the differences in POMS scores and delta band clustering coefficient between active taVNS and sham, we conducted exploratory correlations to test the relationships between EC condition delta-band clustering coefficients and changes in depression and tension scores. In the active taVNS stimulation group, depression domain difference score was positively correlated with clustering coefficient in left frontal (LF), left central (LC), left parietal (LP), and left occipital (LO) regions in the delta band (LF: r = 0.452, *p* = 0.023; LC: r = 0.443, *p* = 0.025; LP: r = 0.438, *p* = 0.027; LO: r = 0.471, *p* = 0.018). No other correlations were found between the tension or depression domains and clustering coefficient in the delta band for the active or sham group.

### 3.5 Secondary analyses

#### 3.5.1 Effects of taVNS on mood

Group differences were observed in changes in tension, *F*(1, 38) = 4.69, *p* = 0.037, and depression, *F*(1, 38) = 4.48, *p* = 0.041; however, neither met the Bonferroni-adjusted significance threshold. The active-taVNS group showed an estimated reduction in depression (mean change = −1.65, SE = 0.45, 95% CI [−2.56, −0.74]) compared with the sham group (mean change = −0.30, SE = 0.45, 95% CI [−1.21, 0.61]). The estimated between-group difference in change in depression was −1.35 points (95% CI, [−2.64, −0.06]). The active-taVNS group also showed an estimated reduction in tension (mean change = −1.75, SE = 0.57, 95% CI [−2.90, −0.60]) compared with the sham group (mean change = 0.00, SE = 0.57, 95% CI [−1.15, 1.15]). The estimated between-group difference in tension change was −1.75 points (95% CI [−3.39, −0.11]).

#### 3.5.2 Effects of taVNS on heart rate variability

Because of technical problems during data collection, HRV data were unavailable for three participants (two active and one sham; all female), resulting in a final HRV sample of 39 participants. Of 117 possible observations across the three timepoints, one additional post-taVNS observation was missing, yielding 116 observations for analysis. There was no clear evidence of a difference between the active and sham groups at the pre-stimulation baseline (*z* = −1.24, *p* = 0.214). Relative to baseline, HRV did not show clear changes during stimulation (*z* = −0.14, *p* = 0.889) or post-stimulation (*z* = 0.55, *p* = 0.582). The estimated group-by-timepoint interaction during stimulation was β = 1.48, 95% CI [−0.36, 3.32], *z* = 1.58, *p* = 0.114. The post-stimulation interaction was β = 0.26, 95% CI [−1.63, 2.15], *z* = 0.27, *p* = 0.786. Both intervals included zero and were relatively wide, indicating uncertainty about the direction and magnitude of the interaction effects.

## 4 Discussion

Non-invasive modulation devices such as taVNS have shown promise for the improvement of cognitive processes. However, to date, most of these studies have focused on narrow learning paradigms (verbal memory, emotion regulation) (Kaan et al., 2021; De Smet et al., 2021), or those exhibiting cognitive deficits, such as in dementia and aging (Wang et al., 2022; Zhou et al., 2022). Overall, findings from this study offer preliminary evidence that acute taVNS may influence executive and neurophysiological outcomes. The adjusted models did not provide clear evidence of taVNS group differences, and the estimated effects were sensitive to covariate specification. Nevertheless, the direction and magnitude of selected estimates may warrant further investigation. Replication in a larger trial with prespecified outcomes, a limited set of theoretically justified covariates, and adequate power to estimate clinically meaningful effects is needed before firm conclusions can be drawn.

Unadjusted analyses suggested possible group differences in BrainHQ and delta correlation coefficient. Active taVNS led to improved BrainHQ performance in tasks assessing inhibitory control, executive functioning, and cognitive flexibility compared with sham. Because BrainHQ utilizes a dynamic algorithm that adjusts task difficulty based on the participant’s moment-to-moment performance, this finding suggests that taVNS facilitates performance in an adaptive cognitive-training context. In BrainHQ, task difficulty is modulated through stimulus presentation speed, perceptual complexity, working memory load, distractor interference, response selection demands, and decision complexity to maintain an optimal level of challenge. This adaptive approach is designed to maximize engagement of neural systems underlying executive function by maintaining performance near an individual’s capacity, thereby continuously taxing cognitive control processes rather than allowing performance to plateau. This dynamic adaptation engages multiple cognitive processes simultaneously and may make BrainHQ sensitive to detecting subtle cognitive changes during taVNS.

Unadjusted models also showed decreased delta-band connectivity during active taVNS relative to sham taVNS, which may reflect a shift towards a more differentiated and efficient cortical network organization, consistent with the notion that vagal modulation facilitates flexible reconfiguration of large-scale brain networks (Frangos and Komisaruk, 2017). Given that EEG data were collected during the *latter part of the taVNS stimulation period*, it is likely that the observed effects combine both acute and residual effects of the preceding taVNS session. Delta-band oscillations (~1–4 Hz) are markers of lower arousal states, large-scale network synchrony, or a more immature or less efficient coordination state of cortical networks (e.g., during deep sleep or low-arousal conditions) and are typically linked to large-scale network coordination, cognitive control, and top-down regulation (Harmony, 2013). A recent closed-loop auricular VNS study during sleep found that targeting the rising-phase of delta waves produced a reduction in delta-power, indicating a modulation away from default low-arousal/low-engagement states (Anzolin et al., 2023). In the context of awake executive function performance, a reduction in delta connectivity (especially in left-hemisphere networks) may reflect a down-regulation of broadly synchronized low-frequency connectivity and a concomitant sharpening of higher-frequency, more focal, functionally specific connectivity. In other words, active taVNS may push the brain toward a higher state of cognitive readiness, reducing reliance on large-scale delta synchrony and enabling more targeted, efficient neural communication. This interpretation aligns with prior EEG and fMRI studies suggesting that taVNS optimizes neural efficiency by modulating the fronto-parietal network that is involved in executive control (Li et al., 2025; Han et al., 2024). However, the absence of a significant direct association between EEG connectivity metrics and behavioral improvements supports the notion that taVNS modulates cortical dynamics, but whether delta-band modulation directly mediates executive-function enhancement remains unknown. It is also important to note that these between-group differences do not take into account potential inter-individual differences in baseline neurophysiological characteristics that may have contributed to the error variance.

Unlike taVNS enhancement of BrainHQ performance, our results did not find a generalized enhancement of executive function. There were no significant differences between active and sham on MID, standard Stroop, or NIH Toolbox measures. Because these measures have static difficulty over the stimulation period, they may not capture momentary changes in neural state induced by taVNS; in contrast, the adaptive BrainHQ tasks may have been more sensitive to transient modulation. It is also likely that, because our healthy-adult sample performed near ceiling on the static tasks, our non-adaptive assessments lacked sufficient sensitivity. These divergent effects of taVNS on adaptive vs. static cognitive performance indicate that taVNS-mediated cognitive benefits may depend on task demand and complexity. Recent literature supports this distinction. Mena-Chamorro et al. (2025) showed that taVNS effects on cognitive flexibility emerge specifically under high task complexity. Similarly, Gálvez-García et al. (2026) demonstrated that taVNS modulates inhibitory control optimally during high motor task complexity, and Sommer et al. (2023) found preferential modulation of cognitive control mechanisms during demanding multitasking environments. Finally, our analysis revealed unexpected, although nonsignificant, patterns in working-memory performance and punishment-avoidance trials of the MID task. Participants in the sham condition performed slightly better than those in the active condition on the NIH Toolbox working-memory subdomain task. In addition, participants in the active condition responded more slowly on punishment-avoidance trials than on neutral trials, the opposite of the expected pattern, whereas participants in the sham condition performed as expected. Because these differences were not statistically significant, they should be interpreted with caution and require replication in a larger, adequately powered sample.

We did not observe a significant effect of stimulation on HRV. Although HRV has often been used as an indicator of vagal activity, our findings align with the meta-analysis by Wolf et al. (2021), which found no significant effect of taVNS on vagally mediated HRV. Recent examples of this central-peripheral dissociation include Gálvez-García et al. (2026), who reported behavioral improvements in inhibitory control while being unable to find multiple target HRV changes, and Sommer et al. (2023) who found increased integrative processing but again no physiological changes. In contrast to these studies with cognitively demanding tasks, those that have found taVNS-induced HRV increases typically use prolonged resting-state stimulation protocols (Clancy et al., 2014; Lamb et al., 2017). Taken together, these findings suggest that HRV effects may depend strongly on stimulation context, task demands, and methodological factors. Because auricular taVNS primarily engages afferent rather than efferent vagal pathways, its effects may be mediated predominantly by central mechanisms (e.g., locus coeruleus-noradrenergic and GABAergic modulation) without producing detectable peripheral cardiac changes.

Our findings did not demonstrate a significant correlation between BrainHQ performance and HRV metrics suggesting that the cognitive benefits of taVNS may not rely solely on canonical autonomic vagal–parasympathetic modulation (e.g., increased HRV, decreased heart rate, improved vagal tone). Anatomically, auricular taVNS primarily engages afferent rather than efferent vagal pathways (Peuker and Filler, 2002); thus, the utility of HRV as a reliable biomarker for these central effects has been debated (Burger et al., 2020). Furthermore, methodological factors in our study, including the demanding behavioral tasks, lack of respiratory control, and use of an earlobe sham, likely limited HRV sensitivity. This central-peripheral dissociation is supported by recent work demonstrating that while taVNS can improve autonomic regulation (e.g., HRV intercorrelations), these peripheral changes do not necessarily translate into behavioral performance differences (Drost et al., 2025). Instead, our findings point to pathways beyond simple autonomic modulation, driven primarily by central neuromodulatory systems (e.g., LC-NE and GABAergic activation), structural connectivity changes, and adaptive neuroplastic processes. For example, VNS has been shown to alter synaptic-density markers (SV2A) and widespread neural-metabolic function even with unilateral stimulation (Binda et al., 2025). Thus, our results reinforce the notion that cognitive enhancement via taVNS is mediated by central network reconfiguration and neuroplasticity rather than solely peripheral cardiac or vagal-tone changes (von Wrede et al., 2022).

## 5 Limitations and conclusions

The effects on BrainHQ and delta clustering coefficient were not robust to covariate adjustment and should therefore be regarded as exploratory and hypothesis-generating. It is also important to note that, although the multivariate models showed a significant effect of taVNS on cognitive performance and EEG, *post hoc* univariate analyses were significant only at the uncorrected threshold and did not remain significant after Bonferroni correction. Accordingly, these findings should be considered exploratory and interpreted cautiously. In addition, the absence of a pre-stimulation EEG baseline limits interpretation of the EEG findings to between-group differences during the later stimulation period and precludes distinguishing the immediate effects of renewed stimulation from residual effects of the preceding taVNS-paired cognitive training session. Nevertheless, the findings highlight the promise of taVNS for cognitive enhancement in an adaptive context, in which transient taVNS-induced changes in attention, cognitive flexibility, processing speed, and inhibitory control may be more likely to influence ongoing performance. In contrast, fixed-difficulty assessments may have failed to capture these subtle improvements because task demands remained constant regardless of changes in cognitive capacity. The observed reduction in delta connectivity, particularly in the left hemisphere, provides mechanistic plausibility and encourages further optimization of stimulation parameters (e.g., side, frequency, timing, and dose) and individual-difference modeling (e.g., baseline connectivity and neuromodulatory responsivity). Because taVNS is thought to facilitate synaptic plasticity through activation of the nucleus tractus solitarius and locus coeruleus, leading to increased norepinephrine and acetylcholine release, pairing stimulation with an adaptive training paradigm may maximize neuromodulatory effects by repeatedly engaging task-relevant cortical networks. Thus, the observed improvements in BrainHQ performance and reductions in delta-band connectivity may reflect enhanced neural efficiency emerging from the interaction between vagally mediated neuromodulation and continuously adaptive cognitive demands.

## Data Availability

The raw data supporting the conclusions of this article will be made available by the authors, without undue reservation.

## Footnotes

1
www.brainhq.com
